# Peri-transplant use of immune checkpoint inhibitors in hepatocellular carcinoma: a transplant oncology perspective on safety, timing, post-transplant management, and future directions

**DOI:** 10.3389/fonc.2026.1826765

**Published:** 2026-05-28

**Authors:** Maen Abdelrahim, Abdullah Esmail, Bandar Al-Judaibi, Ola Khorshid, Bhoori Sherrie, Vincenzo Mazzaferro

**Affiliations:** 1 Section of Gastrointestinal (GI) Oncology, Houston Methodist Cancer Center, Houston, TX, United States; 2 Department of Medicine, Weill Cornell Medical College, New York, NY, United States; 3 Faculty of Medicine, The University of Jordan, Amman, Jordan; 4 Organ Transplant Center of Excellence, King Faisal Specialist Hospital and Research Centre, Riyadh, Saudi Arabia; 5 Medical Oncology Department, National Cancer Institute (NCI), Cairo, Egypt; 6 Department of Oncology, University of Milan, Milan, Italy; 7 Gastro-Intestinal Surgery and Liver Transplantation Unit, The Istituto Nazionale dei Tumori (National Cancer Institute) of Milan, Milan, Italy

**Keywords:** Hepatocellular carcinoma, immune checkpoint inhibitors, liver transplantation, Milan criteria, transplant oncology, UCSF criteria

## Abstract

Immune checkpoint inhibitors (ICPIs) targeting the programmed cell death protein 1 (PD-1)/programmed death-ligand 1 (PD-L1) and cytotoxic T-lymphocyte associated protein 4 (CTLA-4) pathways have transformed systemic therapy for advanced hepatocellular carcinoma (HCC), prompting exploration of their neoadjuvant role in downstaging tumors to meet the liver transplantation (LT) criteria. While ICPIs have demonstrated impressive tumor responses and survival benefits in non-transplant settings, their peri-transplant application is limited by the substantial risk of acute graft rejection due to immune activation against the allograft. Retrospective data indicate rejection rates of approximately 18%–26% pre-LT and 28%–37% post-LT, with fatal outcomes in a subset of cases. Optimal washout periods (typically ≥50–90 days, ideally ~3 months), PD-L1 graft expression, and intensified immunosuppression appear to mitigate but not eliminate the risk. Living donor LT offers scheduling advantages for precise timing. Post-transplant ICPI use remains high-risk and requires individualized decision-making. Emerging biomarkers (e.g., graft PD-L1, ctDNA, and tumor mutational burden) and surveillance strategies hold promise for risk stratification. This review examines the safety profile, timing considerations, class-specific differences, post-LT palliative applications, and innovations needed for the safe incorporation of ICPIs into the transplant oncology protocols for HCC.

## 1 Introduction and concepts of downstaging

**Figure 1:**
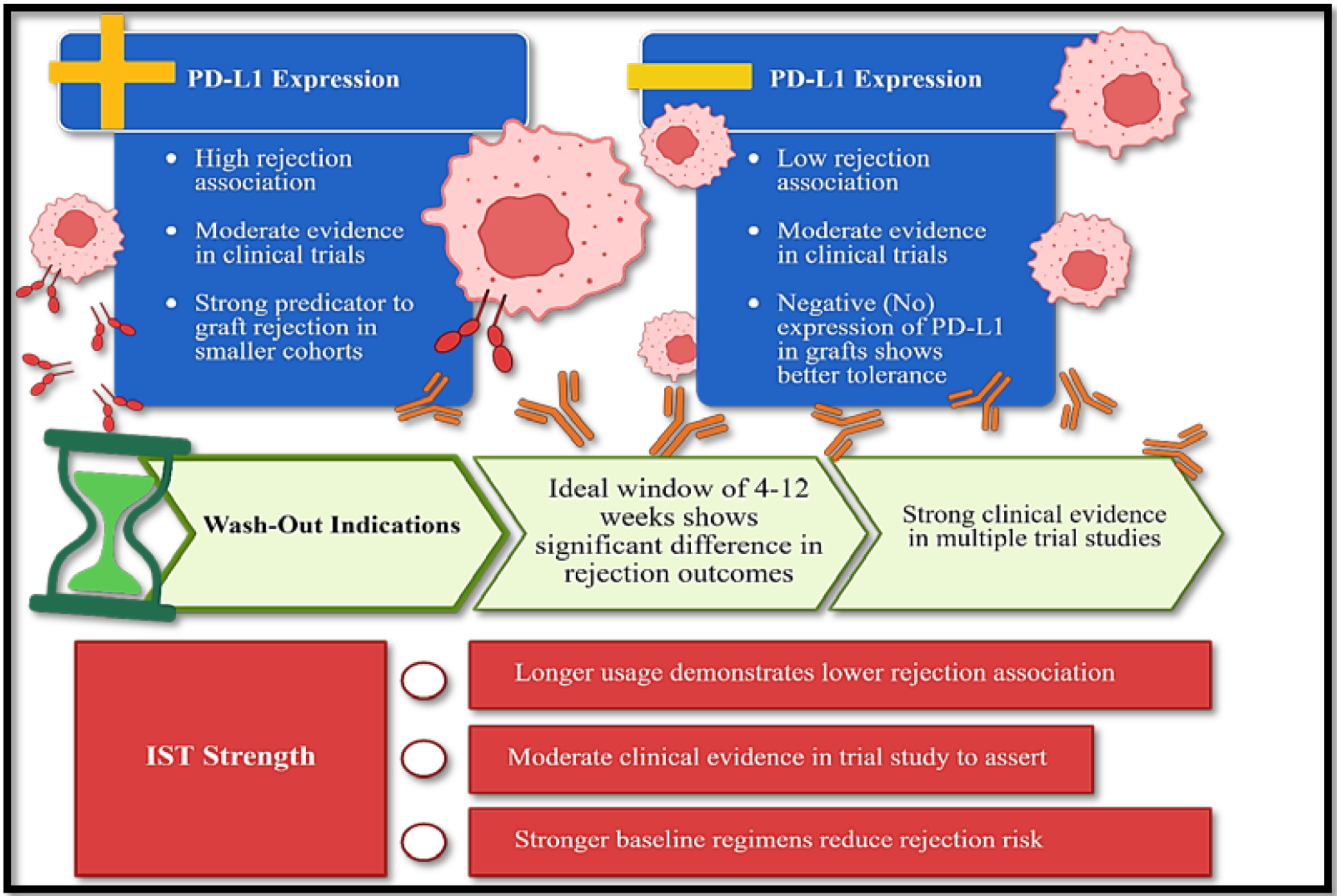
Biomarkers and predictive factors for graft rejection following immune checkpoint inhibitor (ICPI) therapy. *PD-L1*, programmed cell death-ligand 1; *IST*, immunosuppressant therapy.

A unifying conceptual framework for peri-liver transplantation (LT) immune checkpoint inhibitor (ICPI) use is the dynamic balance between antitumor immunity and allograft tolerance, viewed as a temporally regulated immune process. This model consists of three sequential phases: 1) pre-LT immune activation by ICPIs to achieve tumor control and downstaging; 2) a deliberate washout period that allows partial resolution of immune activation while minimizing persistent T-cell effector function; and 3) post-LT intensification of immunosuppression to restore graft tolerance while preserving antitumor memory responses. This framework integrates the key clinical variables, i.e., optimal timing, rejection risk, graft programmed death-ligand 1 (PD-L1) expression, and individualized patient selection, into a single cohesive narrative that guides decision-making throughout the transplant journey (**Figure 1**).

Downstaging refers to the application of locoregional therapy (LRT) for malignancies that exceed the established transplant criteria, the aim of which is to diminish the tumor burden to allow transplantation. This strategy acknowledges that many patients may benefit from transplantation even if they do not strictly fulfill the rigorous Milan criteria (MC) or alternative criteria. However, downstaging raises concerns about increased recurrence risks in patients who initially did not meet the listing criteria.

According to the revised Barcelona Clinic Liver Cancer (BCLC) guidelines, patients classified as BCLC-B (intermediate stage) are stratified into three distinct cohorts. The first subgroup includes those meeting the extended LT criteria, which surpass the minimum requirements, for whom LT is recommended as the primary modality. The University of California, San Francisco (UCSF) criteria, which are well-established within the extended LT framework, stipulate eligibility based on either a solitary tumor ≤6.5 cm or a maximum of three tumors with the largest measuring ≤4.5 cm and a cumulative tumor diameter ≤8 cm. A recent multicenter study evaluating the efficacy of living donor liver transplantation (LDLT) demonstrated no statistically significant difference in the overall survival (OS) rates at 1, 5, and 10 years between cohorts adhering to the MC and UCSF criteria, reporting survival rates of 90.9%, 78.5%, and 64.1% for MC *versus* 88.6%, 73.5%, and 69.4% for UCSF, respectively (*p* = 0.85).

The second cohort encompasses patients with diffuse, infiltrative bilobar tumors who are recommended for systemic therapy. The third subgroup consists of patients with well-defined nodules, maintained portal flow, and targeted vascular access, for whom transarterial chemoembolization (TACE) is advised. Should successful downstaging occur in this subgroup, LT becomes a feasible option as this demographic is identified by revised BCLC standards as the most suitable candidates for downstaging.

According to the European Association for the Study of the Liver (EASL) guidelines, patients in the first cohort, who meet extended criteria such as the UCSF, are considered beyond MC but within the extended criteria, making them eligible for LT without downstaging. Patients in the third cohort, who exceed both the MC and extended criteria initially, may achieve LT eligibility through successful downstaging to within the MC or the extended criteria (such as UCSF), aligning with the endorsement of EASL of downstaging for carefully selected BCLC-B patients.

**Figure 2:**
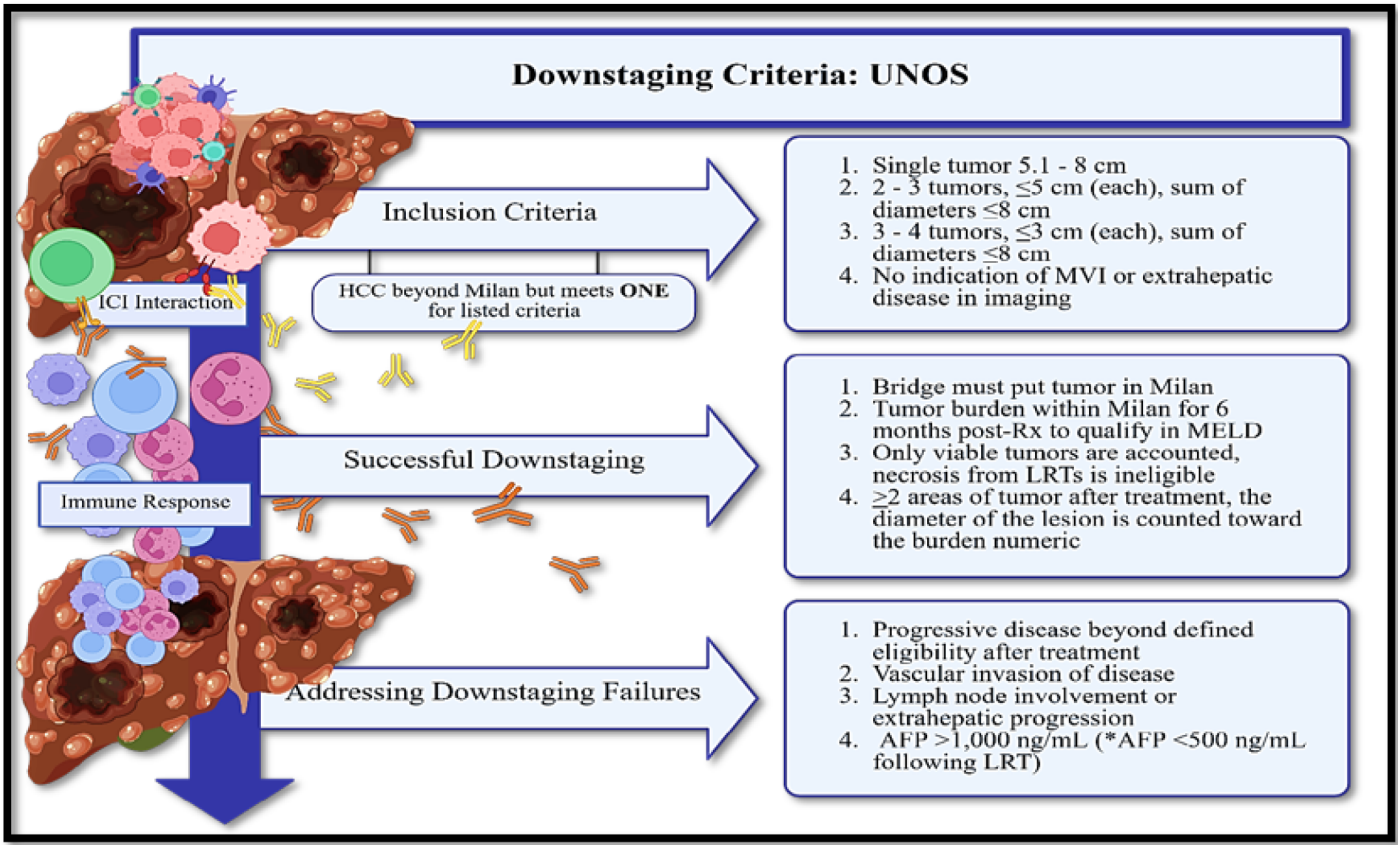
United Network for Organ Sharing (UNOS) downstaging criteria for liver staging in the transplant setting. *MVI*, microvascular invasion; *MELD*, model for end-stage liver disease; *LRT*, locoregional therapy; *AFP*, alpha-fetoprotein.

The United Network for Organ Sharing downstaging (UNOS-DS) strategy, which is depicted in **Figure 2**, revised the tumor size and quantity criteria based on the UCSF LT criteria, permitting larger tumors or an increased number of tumors with proportionately smaller dimensions. This strategy outlines three criteria for successful downstaging: 1) downstaging to the MC; 2) disease stability for a minimum of 6 months; and 3) alpha-fetoprotein (AFP) levels <500 ng/ml when neoadjuvant AFP is >1,000 ng/ml.

Multiple studies have substantiated the clinical efficacy of LT following the UNOS-DS protocol. Mehta et al. analyzed 3,698 patients who underwent LT, revealing no significant difference in the 3-year post-transplant survival rates between the MC and UNOS-DS groups (83.2% *vs*. 79.1%, *p* = 0.17). Notably, they observed that the post-LT mortality risk was elevated in regions with shorter wait times (under 9 months from successful downstaging to transplantation) compared with those with longer wait times [hazard ratio (HR) = 3.1, *p* = 0.005] and in patients with AFP levels ≥100 ng/ml at transplantation (HR = 2.4, *p* = 0.009). Tabrizian et al. found that the 10-year OS rates post-LT were 61.5% for MC and 52.1% for UNOS-DS (*p* < 0.001), with corresponding 10-year recurrence rates of 13.1% for MC and 20.6% for UNOS-DS (*p* < 0.001). These findings suggest that the UNOS-DS approach may be beneficial for prioritizing LT candidacy, given the favorable 10-year post-transplant outcomes.

Factors associated with poor recurrence-free survival (RFS) in the downstaged cohort included a neutrophil-to-lymphocyte ratio (NLR) exceeding 5 at LT [odds ratio (OR) = 2.04, 95% confidence interval (CI) = 1.34–3.09, *p* < 0.001] and the largest viable tumor on explant pathology measuring greater than 5 cm (OR = 2.18, 95%CI = 1.34–3.56, *p* = 0.002), independent of the pretreatment tumor characteristics. Clinical predictors of downstaging failure included the presence of more than three tumors at diagnosis (OR = 2.34, 95%CI = 1.22–4.50, *p* = 0.01), tumor size exceeding 7 cm at diagnosis (OR = 2.62, 95%CI = 1.20–5.75, *p* = 0.02), and an AFP response of at least 20 ng/ml with less than 50% reduction (OR = 1.99, 95%CI = 1.14–3.46, *p* = 0.02) (1). Mehta et al. highlighted the significance of tumor markers in predicting downstaging failure, positing that a pretreatment AFP level exceeding 1,000 ng/ml serves as a superior indicator compared with inadequate liver function (HR = 1.6, *p* < 0.001) (2). Thus, there is an urgent need for improved therapeutic strategies for this high-risk population to facilitate effective downstaging while factoring in advancements in tumor biology, including NLR and other pertinent metrics. This review offers a unique transplant oncology view by integrating the most recent data, providing a dedicated emphasis on precise washout timing and the scheduling advantages of LT, and incorporating the newly developed unifying conceptual framework and mechanistic insights.

### 1.1 Expansion of the downstaging criteria: BCLC-B (beyond UNOS-DS)

“All-comers” (AC) refers to patients whose tumor burden initially exceeds the UNOS-DS criteria. The “all-comers” downstaging AC-DS protocol specifies the inclusion of patients with HCC who exceed the UNOS-DS requirements but are still classified within BCLC-B. The concept of successful downstaging in the AC-DS protocol is similar to that of the UNOS-DS protocol. However, research on the clinical outcomes of the AC-DS protocol is less extensive than that of UNOS-DS treatment.

Research conducted by Mehta et al. revealed a significant difference in the 3-year post-LT OS rates between the MC and ACDS groups (83.2% *vs*. 71.4%, *p* = 0.04). Natarajan et al. examined 311 patients who underwent LT (229 in the UNOS-DS group and 82 in the ACDS group) and reported 3-year OS rates of 69% in the UNOS-DS cohort and 58% in the ACDS cohort (*p* = 0.05). The 3-year OS rates post-LT were not significantly different between the two groups (91% *vs*. 81%, *p* = 0.67).

In addition, the 3-year OS rate following LRT in patients with advanced cirrhosis and decompensation who lacked high-risk characteristics (AFP >500 ng/ml or Child–Pugh class B/C cirrhosis) was 67.7%. In contrast, it was only 29.9% for patients with one or two high-risk factors (*p* = 0.003). LT may apply to carefully selected AC-DS patients with favorable tumor biology, provided effective downstaging exists.

Nevertheless, the outcomes in the AC-DS population remain modestly inferior to those achieved with standard UNOS-DS or MC patients, underscoring the higher inherent biological risk of this “all-comers” cohort. The observed survival gap highlights the critical importance of rigorous patient selection based on dynamic tumor biology, particularly sustained radiological response, AFP normalization or a marked decline, and absence of high-risk features such as Child–Pugh B/C decompensation. In current transplant oncology practice, AC-DS should be reserved for highly selected individuals who demonstrate clear downstaging and favorable biology after LRT or systemic therapy. The integration of modern neoadjuvant approaches, including ICPIs, may further refine this strategy; however, prospective validation within multicenter consortia is essential before broader implementation.

### 1.2 Expansion of the downstaging criteria: BCLC-C and portal vein tumor thrombosis

Historically, portal vein tumor thrombosis (PVTT) has been considered a contraindication for LT. However, recent studies have indicated that deceased donor liver transplantation (DDLT) and LDLT offer significant clinical advantages. For instance, Yu et al. analyzed 961 patients who underwent LT (3), classifying them based on their status concerning the MC: 489 patients within the criteria, 296 beyond the criteria without PVTT, 83 with type 1 PVTT, and 93 with type 2 PVTT. The study found that the 5-year OS rate for patients with type 1 PVTT (78.3%) was not significantly different from those within the MC (79.1%, *p* = 0.062). Furthermore, it was superior to the survival rate of patients beyond the MC without PVTT (50.0%, *p* = 0.012).

The study highlighted that the OS and RFS rates for patients with PVTT and AFP levels ≤100 ng/ml were significantly better compared with those for individuals beyond the MC or for PVTT patients with AFP levels >100 ng/ml (*p* < 0.01). Notably, there was no substantial difference in the OS rates between patients with PVTT and AFP levels ≤100 ng/ml and those within a matched cohort (*p* = 0.065). In another study by Soin et al. involving 405 LT patients (382 without PVTT and 23 with downstaged PVTT), the 5-year OS and RFS rates for those with downstaged PVTT were not significantly different from those without PVTT (57% *vs*. 65%, *p* = 0.06; 66% *vs*. 51%, *p* = 0.33) (4). Moreover, the 1-year OS rate of patients with downstaged PVTT was significantly higher than that of PVTT patients undergoing sorafenib monotherapy (82% *vs*. 0%, *p* < 0.001). Serenari et al. conducted a prospective pilot study that evaluated the clinical outcomes in five HCC patients with PVTT who received DDLT after successful downstaging with transarterial radioembolization (TARE). The results showed that the 5-year OS rate for the DDLT group was significantly better than that of the observation group (60% *vs*. 0%, *p* = 0.03). These findings suggest that LT should be carefully considered for highly select patients with PVTT following effective downstaging.

These promising results derive predominantly from retrospective and small prospective series with inherent selection bias and heterogeneous downstaging regimens. In contemporary transplant oncology, LT can be reasonably considered in highly select patients with PVTT once effective downstaging is achieved, particularly when AFP is low and sustained radiological response is documented. However, such decisions demand rigorous multidisciplinary approaches (MDA), careful assessment of the macrovascular invasion extent, and individualized risk–benefit analysis. The integration of modern neoadjuvant systemic therapies, including ICPIs, holds potential to further expand safe candidacy; however, prospective validation within multicenter protocols is essential before this approach can be broadly adopted.

### 1.3 Expansion of the downstaging criteria: the role of a multidisciplinary approach in the era of immuno-oncology therapy

The implementation of MDA has been demonstrated to significantly enhance the survival outcomes in patients with HCC. El Dahan et al. conducted a meta-analysis encompassing 12 trials with a total of 15,365 patients with HCC, revealing that MDA is associated with improved OS, yielding a HR of 0.63 with a 95%CI of 0.45–0.88. Furthermore, Sinn et al. evaluated a cohort of 6,619 patients with HCC, of whom 738 received MDA care. The findings indicated markedly improved 5-year survival rates for patients under MDA (71.2%) *versus* those who were not (49.4%), with a *p*-value <0.001. This approach is especially beneficial for patients classified as BCLC-B or BCLC-C with AFP levels exceeding 200 ng/ml, identifying them as potential candidates for downstaging.

Effectively executing downstaging, especially the maintenance of tumor biology stability, has been shown to enhance survival and prognosis post-LT, even among those whose initial tumor burden surpasses the extended LT criteria (such as UNOS-DS, AC-DS, and PVTT). Thus, employing advanced treatment strategies, particularly in conjunction with MDA, optimizes the probability of achieving successful downstaging prior to LT.

Recent advancements in systemic therapies for HCC, particularly those involving ICPIs, have yielded encouraging results (5–7). The pivotal IMbrave150 trial demonstrated that the combination of atezolizumab and bevacizumab resulted in superior OS compared with sorafenib, with the median OS reported at 19.2 months *versus* 13.4 months (HR = 0.66). Complementarily, the HIMALAYA trial showcased improved OS with the durvalumab plus tremelimumab combination therapy, yielding median values of 16.4 months *versus* 13.8 months (HR = 0.78) compared with sorafenib.

Integrating immuno-oncology (IO) therapies with LRT such as TACE, TARE, and radiation therapy (RT) may further bolster the downstaging success rates. This combination is particularly compelling as LRT can elicit the release of tumor-associated antigens and activate antigen-presenting cells, resulting in an immunologically responsive tumor microenvironment. Kudo et al. studied 110 patients with unresectable or TACE-ineligible BCLC-B HCC subjected to atezolizumab and bevacizumab therapy, reporting that 25% of the cohort achieved complete response and were eligible for curative-intent interventions post-therapy. Moreover, Ding et al. executed a meta-analysis covering 1,174 patients across 19 trials, which indicated a significant enhancement in outcomes for the group receiving combined cellular immunotherapy and LRT, predominantly TACE (8).

Taken together, these data underscore that MDA significantly improves survival in complex HCC cases and should be considered standard practice for patients undergoing downstaging, especially those with advanced BCLC-B or BCLC-C disease. The synergy between LRTs and modern systemic agents, most notably ICPI combinations, offers a compelling strategy to enhance downstaging success and post-transplant outcomes. Nevertheless, the optimal integration of these modalities requires prospective trials to define the ideal sequencing, patient selection criteria, and long-term impacts on recurrence and graft survival. In the current era of IO, a truly MDA is no longer optional but rather the cornerstone of successful transplant oncology care.

## 2 Safety concerns and risk of peri-transplant ICPI use

The exact processes underlying immune-related adverse events (irAEs) remain unclear; nevertheless, they are believed to arise from the bystander effects of activated T cells, which align with the mechanisms of action of ICPIs (9, 10). Tumors characterized by inflammation with cytotoxic T cells before treatment undergo further inflammation and tumor cell mortality after the application of ICPIs. Similarly, an organ exhibiting subclinical inflammation may undergo significant, clinically observable inflammation after the elimination of crucial negative regulators of T-cell activity. Nonetheless, the mechanisms that result in particular toxicities in various patients, as well as the relationship between toxicity and therapeutic response, remain inadequately understood.

Subsequent study emphasizes the relationship between T cells and irAEs, underscoring the importance of the gut flora. Marked disparities in microbial diversity and composition have been observed between melanoma patients who respond to anti-PD-1 therapy and those who do not. Many studies indicate that certain species may be more abundant in responding patients relative to non-responders (11, 12). Studies employing fecal microbiota transplants in murine models have provided significant molecular insights. Mice that had fecal transplants from patients responsive to anti-PD-1 antibodies exhibited an increased density of CD8 T cells within the tumor tissue. Mice who had fecal transplants from responders exhibited elevated levels of CD8 T cells in the gut compared with those that received stool from non-responders. A study of 26 patients with metastatic melanoma treated with ipilimumab indicated that individuals with baseline gut microbiota enriched in *Faecalibacterium* and other Firmicutes exhibited improved progression-free survival (PFS), OS, and elevated rates of ICPI-induced colitis compared with patients lacking such enrichment (13). Patients exhibiting a greater abundance of Firmicutes demonstrated a reduced proportion of regulatory T cells and alpha4beta7 integrin-positive CD4 and CD8 T cells relative to those lacking this enrichment. The composition of the microbiome may be associated with both toxicities and responses; however, the significance of certain microbial species is still ambiguous. Additional prospective studies are required. More laboratory research indicates the existence of autoimmune toxicity mechanisms that operate independently of the antitumor response. Swiss Jim Lambert (SJL) mice in a hypophysitis model associated with ipilimumab were administered an immunoglobulin G1 (IgG1) hamster antibody that antagonizes CTLA-4 using a dosage regimen analogous to that employed in humans (14). No infiltration was observed in other organs of treated mice, including the thyroid gland, skin, colon, or liver. No pituitary antibodies were detected in either the pretreatment mice or the control groups. CTLA-4 mRNA expression was identified in the mouse pituitary gland, predominantly in lactotrophic and thyrotrophic cells, but markedly reduced levels were observed in the murine thyroid gland. This research posits that the production of preexisting organ-specific antigens may contribute to the autoimmune toxicity induced by ICPIs, independent of any shared effects associated with antitumor activity.

While these mechanistic insights derived largely from non-transplant melanoma cohorts provide valuable biological context, their direct translation to the peri-transplant HCC setting remains limited. Patients with underlying cirrhosis exhibit altered immune homeostasis, heightened susceptibility to hepatic decompensation, and unique graft tolerance dynamics that are not fully captured by extrahepatic tumor models. In transplant oncology, the critical challenge lies in harnessing antitumor immunity without triggering clinically significant graft rejection. Prospective studies focused specifically on the LT population are therefore essential to delineate which irAE mechanisms are most relevant and to develop tailored mitigation strategies that preserve both oncologic efficacy and graft function

### 2.1 Which ICPI class is safer?

With regard to the safety profiles of ICPIs, the current literature presents limited data to support the relative safety of one ICPI over another, although numerous studies have aimed to address this. Munker et al. examined 14 patients receiving ICPI treatment, finding that only two were administered a CTLA-4 inhibitor (ipilimumab), while the remaining patients received PD-1 or PD-L1 blockers (nivolumab and pembrolizumab) (15). The data were excessively varied to reach definitive conclusions, and it has been asserted that additional criteria, such as age, liver biopsy, and therapy timing, are relevant (16). However, the variability in the data precludes definitive conclusions, and additional parameters such as patient age, liver biopsy results, and therapy timing have been suggested as influential factors. Other case reports noted that 4 out of 10 patients with organ rejection were treated with PD-1 inhibitors, whereas no rejection was observed in two patients who received a CTLA-4 inhibitor such as ipilimumab (17–22). However, additional research is required to draw definitive results.

The existing literature, composed primarily of small case series and heterogeneous retrospective reports, does not permit any firm conclusion regarding class-specific safety in the peri-transplant context. Although isolated observations suggest a potentially lower rejection signal with CTLA-4 inhibitors compared with PD-1/PD-L1 agents, confounding factors including patient age, washout duration, baseline immunosuppression, and tumor burden preclude reliable comparisons. Until larger, adequately powered studies with standardized endpoints are available, no single ICPI class can be preferentially recommended for bridge or downstaging therapy. Clinicians must therefore individualize agent selection based on tumor biology, liver function, and planned transplant timing rather than presumed class differences.

### 2.1 Mechanistic basis of ICPI-induced graft rejection

The PD-1/PD-L1 pathway plays a central role in maintaining allograft tolerance after LT by inducing T-cell exhaustion and limiting effector responses against donor antigens. In the tumor microenvironment, the same pathway enables immune evasion by HCC cells. ICPIs block this inhibitory signaling, thereby restoring CD8^+^ T-cell function and promoting antitumor cytotoxicity. However, this restoration also reactivates alloreactive T cells that recognize donor antigens in the graft, leading to acute cellular rejection. Memory T-cell populations generated during pre-transplant ICPI exposure further amplify this response as they persist even after drug clearance and can rapidly trigger graft-directed immunity upon re-exposure to alloantigens. This mechanistic interplay explains why shorter washout periods and younger recipient age are consistently associated with higher rejection rates. Understanding this balance between antitumor activation and loss of graft tolerance is essential for optimizing the timing and post-transplant immunosuppression strategies in the transplant oncology setting.

This mechanistic framework underscores why shorter washout intervals and younger recipient age consistently emerge as dominant risk factors for rejection. Memory T-cell populations primed during pre-LT ICPI exposure persist long after drug clearance and can be rapidly reactivated upon allograft antigen encounter. In the transplant oncology setting, successful mitigation therefore requires not only adequate washout but also strategic intensification of post-LT immunosuppression and, when feasible, graft PD-L1 assessment. Future research should focus on the modulation of these pathways to uncouple beneficial antitumor immunity from deleterious alloreactivity.

## 3 Pre-transplant (neoadjuvant) ICPI use: evidence, timing/washout, and outcomes

The utilization of ICPIs prior to transplantation has also been scrutinized. Initial reports from Nordness et al. (23) documented the use of nivolumab, a PD-1 inhibitor, in a patient who experienced severe acute hepatic necrosis post-surgery and subsequently died. Similarly, Chen et al. (24) noted a lethal outcome after the administration of toripalimab, another anti-PD-1 antibody, before transplantation. In contrast, a study by Tabrizian et al. (25) involving nine patients treated with nivolumab showed that, while one patient experienced mild acute rejection, there were no severe rejection episodes or graft losses, indicating that some patients may tolerate this treatment better.

One of the primary concerns with pre-transplant ICPI administration is graft rejection, particularly regarding the importance of the PD-1/PD-L1 pathway in graft acceptance. Consequently, several researchers have proposed a minimum washout period following the last ICPI administration, recommending up to 3 months to minimize rejection risks (26–29). In addition, a retrospective study by Beat et al. analyzed over 100 cases and proposed a 50-day washout period for ICPIs prior to transplantation, reporting reduced rejection rates while maintaining oncologic efficacy, further supporting the feasibility of shorter washout periods in select patients (30). With nivolumab and atezolizumab having a half-life of approximately 27 days, this timeframe allows plasma concentrations to fall below relevant levels (31). However, the current literature consists mainly of case reports and short series with inconsistent findings. In the study by Tabrizian et al. (25), four patients received their last dose of nivolumab within 14 days prior to transplantation and did not exhibit signs of rejection (32, 33).

In contrast, recipients from Nordness and Chen et al. suffered fatal acute hepatic necrosis after receiving their last ICPI doses at 8 and 93 days before transplantation, respectively (23, 24). These outcomes suggest that additional factors beyond the duration since the last ICPI administration may influence graft acceptance, indicating that the serum half-life alone may not be sufficient to establish an optimal washout period. Notably, drug-related side effects and antitumor effects have been observed to persist long after administration of the drugs, further complicating treatment protocols (34).

ICPIs operate by altering the regulatory processes of the immune system, resulting in a notable incidence of irAEs that mimic autoimmune illnesses and can affect nearly all organ systems. Severe irAEs are immune toxicities that require systemic corticosteroid or immunomodulatory intervention and generally lead to the discontinuation of ICPI therapy. Patients diagnosed with HCC experience high-grade irAEs at rates similar to those of other cancer types, ranging from 10% to 20%, depending on the specific ICPI regimen utilized (35–38). Individuals diagnosed with HCC constitute a vulnerable population regarding irAEs due to preexisting liver diseases. Given that over 90% of patients with HCC have preexisting cirrhosis (39), there are apprehensions regarding the potential of irAEs to induce hepatic decompensation. Hepatic decompensation denotes a substantial decline in liver function, often linked to complications stemming from liver cirrhosis. This syndrome may be triggered by substantial inflammation in other organs or by direct hepatic inflammation, such as ICPI hepatitis, resulting in diminished liver function and portal hypertension. ICPI hepatitis is a common and severe immune-mediated side effect, impacting 2%–10% of patients, contingent upon the therapy protocol. Moreover, ICPI hepatitis is the irAE most prone to present as high grade upon onset (40–42). Identifying the underlying causes of elevated liver tests in patients with cirrhosis and liver cancer poses significant challenges. A study including 375 individuals treated with atezolizumab and bevacizumab revealed an 11.4% prevalence of ICPI hepatitis. In addition, 6%–9% of patients in phase III clinical trials necessitated corticosteroid treatment for ICPI-induced hepatitis (35, 36, 43). Clinical trial data suggest the possibility of hepatic decompensation, with documented instances of ascites (6%–7%), portal hypertension-related hemorrhage (1%–4%), and encephalopathy (1%–3%). While these trials did not investigate the hypothesized causation, in particular the temporal relationship between an irAE and a decompensating incident, and considering that some of the reported decompensating events likely resulted from the natural progression of liver disease, the inclusion of only patients with optimal baseline liver function (Child–Pugh class A) across all three trials suggests that the emergence of irAEs may precipitate hepatic decompensation in a particular subset of patients (44). Moreover, it indicates that decompensation may occur more frequently in practical situations requiring immunotherapy for patients with severe liver disease (45). Results from a limited clinical trial and a meta-analysis indicate that managing patients with Child–Pugh class B illness is feasible, with comparable rates of irAEs to those observed in patients with Child–Pugh A cirrhosis (46, 47). The absence of decompensation data and the finding that individuals with Child–Pugh B cirrhosis exhibit reduced OS suggest that additional research is necessary (46, 48).

The association between the start of irAEs and the response to ICPI is apparent, although the details of this connection remain unclear. Current study aims to differentiate between therapeutic efficacy and harmful consequences. A phase II experiment was executed to validate the approach, integrating high-dose ipilimumab with or without sargramostim (granulocyte–macrophage colony-stimulating factor, GM-CSF) in patients diagnosed with metastatic melanoma (49). Patients administered GM-CSF demonstrated reduced toxicity and enhanced longevity relative to those receiving solely high-dose ipilimumab, while the response rates were similar in both cohorts. The mechanism underlying this impact remains incompletely elucidated; however, a phase III trial investigating ipilimumab and nivolumab, with or without GM-CSF (NCT02339571). Interleukin 6 (IL-6) is a cytokine that may serve as an additional target. A recent study examined RNA from patient-matched normal colonic tissue and colitis tissue associated with irAEs (50). Variations in the gene expression were analyzed between normal and colitis tissues, as well as baseline and on-treatment tumor biopsies from patients who responded to ipilimumab *versus* those who did not. In tissue from patients with irAE-induced colitis, *IL-6* exhibited the most pronounced differential overexpression relative to normal colonic tissue. *IL-6*, along with several other genes that are differentially elevated in colitis tissue from patients, did not exhibit a substantial increase in responsive tumors. *IL-6* was identified as the gene that showed differential overexpression in tumor tissue from non-responsive individuals. The researchers inhibited IL-6 alongside CTLA-4 in murine models, resulting in substantial tumor shrinkage that exceeded the results observed in mice treated only with anti-CTLA-4 antibodies. Thus far, there has been no clinical evaluation of anti-IL-6 targeted therapy in conjunction with ICPI. A clinical trial in patients with advanced melanoma has been discontinued. The study investigated the combination of nivolumab and ipilimumab with the alpha4beta7 integrin antagonist vedolizumab and the human chemokine receptor 2 antagonist plozalizumab to assess the possibility for differentiating antitumor activity from autoimmunity (NCT02723006).

A significant correlation exists between autoimmunity and the antitumor effects induced by ICPIs. Contemporary oncology research is increasingly focused on the potential to dissociate these two ICPI components in order to enhance benefits while reducing patient toxicities. irAEs may serve as a clinical biomarker reflecting the response to ICPI, despite their emergence during treatment. The incidence of irAEs linked to ICPIs demonstrates a more robust association with the efficacy of anti-PD-1 and anti-PD-L1 antibodies compared with anti-CTLA-4 antibodies. This may be ascribed to the particular diseases for which each agent has obtained FDA approval, the differing mechanisms of action of the agents, or the duration of treatment (e.g., four doses followed by cessation for anti-CTLA-4 *versus* extended treatment for anti-PD-1 or anti-PD-L1). Numerous questions persist regarding the interaction of irAE features, including location, severity, onset time, management approaches, and the ICPI effectiveness. Further rigorously designed research is necessary to elucidate the impact of the irAE features on ICPI responsiveness in patients.

Recent research works by Houston Methodist have investigated clinical factors that may influence rejection rates, particularly the interval designated as the “washout” period between ICPI administration and LT (51, 52). This period acts as a break between systemic therapies and transplantation, allowing for the modulation of the host immune system to eliminate PD-1 and CTLA-4 binding receptors. Disruption of the immunological B7 pathways may enhance T-cell activity, potentially resulting in T-cell-mediated graft rejection (53).

Several ICPIs, including nivolumab and pembrolizumab as monotherapies, as well as combinations such as nivolumab with ipilimumab or other FDA-approved therapies such as atezolizumab and bevacizumab, have shown substantial improvements in the survival outcomes and overall response rates of patients with unresectable HCC. While the findings indicate the general tolerability of ICPIs, research has reported a wide range of adverse events (AEs), with only 15% of patients with unresectable HCC experiencing AEs that required treatment cessation. However, challenges remain concerning graft rejection and loss.

The application of ICPIs is advancing rapidly within the field; however, the safety of ICPI therapy is still uncertain and requires further investigation. A recent study involving nine patients with HCC who underwent transplantation after receiving nivolumab as a neoadjuvant therapy indicated positive results (25). No significant allograft rejection or loss was noted at a median follow-up of 16 months post-transplantation. In addition, there were no reports of tumor recurrence or mortality during the same median follow-up period. It is important to mention that one patient experienced mild acute rejection due to low tacrolimus levels, but which resolved promptly after adjusting the immunosuppressant dosage. In the explanted livers, nearly one-third of those assessed showed near-complete (>90%) tumor necrosis. Despite these encouraging results, the study concluded that more prospective research on ICPIs in the pre-transplant setting is needed to better understand their appropriate use in patients awaiting LT (**Table 1**).

**Table 1:** Summary of published studies evaluating the use of immune checkpoint inhibitors (ICPIs) prior to liver transplantation (LT), highlighting treatment regimens, interval between last ICPI dose and transplantation, and post-transplant outcomes including rejection, tumor recurrence, and early mortality.

Study	*N*: Number of Patients	ICPI regimen	Interval (ICPI → LT)	Rejection	Recurrence	Mortality
Xu 2024 (54)	25	PD-1-based	40–151 days	3 cases	6 cases	NR
Abdelrahim 2024 (55)	6	Atezo + Bev Nivo + Ipili Nivo	~5 months	None	5 cases	2
Tabrizian 2024 (56)	117	Nivo Atezo + Bev Pembrolizumab Durvalumab + Tremelimumab	13–120 days	7 cases	NR	NR
Guo 2024 (57)	83	Camrelizumab Pembrolizuamb Sintilimab Tislelizumab Nivo Atezo	NR	23 cases	20 cases	5
Liu 2024 (58)	9	Atezo + Bev Nivo + Ipili Nivo Pembrolizumab	~3 months	1 case	NR	NR
Kumar 2024 (59)	1	Atezo + Bev	6 weeks	None	None	No
Tabrizian 2024 (60)	17	Atezo + Bev	41–123 days	2 cases	NR	NR
Giudicelli 2023 (61)	1	Atezo + Bev	6 months	None	None	NR
Ohm 2023 (62)	3	Atezo + Bev Nivo + Ipi	2–7 days	None	None	No
Chouik 2023 (63)	1	Atezo + Bev	1 week	None	None	No
Rudolph 2023 (64)	1	Nivo	55 days	GVHD	None	No
Wang 2023 (65)	16	Nivo Pembrolizumab Sintilimab Camrelizumab	1–184 days	9 cases	5 cases	NR
Dave 2022 (66)	8	Nivo	~105 days	2 cases	NR	1
Kang 2022 (67)	1	Pembrolizumab	138 days	None	None	No
Yin 2022 (68)	1	PD-1 + Lenvatinib	NR	Yes	NR	NR
Abdelrahim 2022 (69)	1	Atezo + Bev	2 months	None	None	No
Aby 2022 (70)	1	Nivo	16 days	Yes	NR	No
Schnickel 2022 (71)	5	Nivo	Variable	1 severe	None	No
Tabrizian 2021 (25)	9	Nivo	4 weeks	Mild	None	No
Sogbe 2021 (72)	1	Durvalumab	90 days	None	None	No
Qiao 2021 (73)	7	Pembrolizumab Camrelizumab + Lenvatinib	~40 days	Yes	NR	NR
Lizaola-Mayo 2021 (74)	1	Nivo + Ipi	8 weeks	None	None	No
Dehghan 2021 (75)	1	Nivo	5 weeks	Graft loss	NR	NR
Chen 2021 (76)	5	Nivo	2–4 months	None	2 cases	NR
Chen 2021 (24)	1	Toripalimab + Lenvatinib	93 days	Severe necrosis	N/A	Yes
Schwacha-Eipper 2020 (28)	1	Nivo	21 weeks	None	None	No
Nordness 2020 (23)	1	Nivo	8 days	Yes	N/A	Yes

*ICPI*, immune checkpoint inhibitor; *LT*, liver transplantation; *PD-1*, programmed cell death protein 1; *Atezo*, atezolizumab; *Bev*, bevacizumab; *Nivo*, nivolumab; *Ipi*, ipilimumab; *NR*, not reported; *GVHD*, graft-*versus*-host disease.

Collectively, the published experience demonstrates that neoadjuvant ICPI use prior to LT is feasible in carefully selected patients when an adequate washout period is observed. However, the preponderance of retrospective case series, variable washout intervals, inconsistent immunosuppression regimens, and heterogeneous reporting of rejection and oncologic outcomes preclude broad generalization. LDLT offers a distinct logistical advantage by allowing precise scheduling of the washout interval; however, even in this setting, the rejection risk is not eliminated. Until robust prospective multicenter trials define optimal patient selection, washout duration, and post-LT immunosuppression protocols, neoadjuvant ICPI should remain restricted to multidisciplinary tumor board discussion and preferably within clinical trial frameworks.

## 4 What staging system is best for determining prognosis post-LT

Determining the best staging system for the assessment of prognosis after LT has become increasingly complex. Currently, the staging and allocation of organs for patients are heavily reliant on imaging investigations. Despite significant advancements in imaging technology, the outcomes frequently do not correlate well with findings from surgical specimens. Preoperative staging may be altered based on the pathological evaluation of the transplanted liver, as many characteristics critical for staging are only revealed post-transplantation. This limitation makes initial assessments unsuitable for determining a patient’s transplantation or organ allocation eligibility.

Research indicates that up to 30% of individuals diagnosed with HCC through preoperative imaging or AFP levels possess alternate histological diagnoses, suggesting the need for downstaging. Moreover, several small HCC lesions identified during pathological assessments often remain undiagnosed before surgery. These lesions are typically minor and have a minimal impact on the OS outcomes. While the decision to proceed with transplantation or to evaluate a patient against the transplantation criteria may initially depend on the imaging stage, the new TNM staging approach incorporates insights from pathological examinations, enhancing its accuracy.

Significant prognostic indicators for HCC in excised livers include the tumor dimensions and the number of tumors present. Additional staging markers, such as microvascular invasion (MVI), satellite nodules, and lymph node metastases, can provide further prognostic insights. Beyond the stage, the characteristics related to tumor grades—such as differentiation, nuclear size, atypia, and mitotic activity—also hold significant prognostic value. The Edmondson and Steiner grading system, which was established in 1954, is frequently referenced in research on prognostic indicators for recurrence. However, a recent consensus statement has proposed a three-point grading system, which merges grades 1 and 2 from the Edmondson–Steiner scheme into a single, well-differentiated grade for reporting lesions in explanted livers.

Furthermore, the incorporation of additional tissue-based investigations, including analyses of novel genetic markers that may delineate aggressive phenotypes, into pre-transplant and post-transplant grading frameworks is gaining traction. While related literature explores these aspects further, they are essential considerations in any serious discussion regarding cancer staging in the context of LT.

In the current era of neoadjuvant immunotherapy, traditional imaging-based staging systems are increasingly insufficient. Pathology-integrated models that incorporate MVI, tumor grade, satellite nodules, and dynamic biomarkers [e.g., AFP response or circulating tumor DNA (ctDNA)] provide superior prognostication. Future staging frameworks must evolve to include treatment–response metrics and molecular features so that transplant candidacy reflects not only the pretreatment tumor burden but also the biological behavior after modern systemic therapies. Until such refined systems are validated, explant pathology remains the gold standard for accurate post-LT risk stratification.

## 5 Post-transplant palliative therapy

Immune therapy has generally been deemed contraindicated for solid organ transplant recipients due to safety concerns, mainly due to an increased risk of allograft rejection in these patients. However, there are documented instances in which specific LT recipients have been administered ICPIs in suitable and distinct contexts. Studies involving LT recipients treated with ICPIs have shown that nearly two-thirds of the allografts were preserved, with a disease control rate of 21% and a total graft rejection rate of 37% (77).

Additional research studying the use of ICPIs post-LT indicates a more hazardous appearance than when administering a neoadjuvant. In addition, recipients undergoing ICPI therapy should be subjected to rigorous monitoring. Montano-Loza et al. (78) introduced a decision table that assists in the application of ICPIs for LT recipients, considering individual immunological risks and potential oncological benefits.

Despite limited data regarding atezolizumab (a PD-L1 antibody) in the transplant context, none of the 10 patients (five pre-LT and five post-LT) treated with atezolizumab experienced graft rejection (64) (**Figure 1**). The safety of peri-transplant atezolizumab administration relative to other ICPIs requires further investigation. Graft PD-L1 expression is proposed as a potential biomarker for patient selection as it may be correlated with rejection probability (34, 79). In a study involving nine recipients with accessible graft biopsies, four exhibited positive PD-L1 staining and experienced graft rejection, while the remaining five, with negative PD-L1 expression, did not experience rejection. Despite the potential of PD-L1 as a selection criterion for ICPI therapy, its prognostic significance pre-transplant is unclear. In cases documented by Nordness et al. and Chen et al., pre-transplant PD-L1 staining was negative. At the same time, post-transplant PD-L1 expression was positive, suggesting that PD-L1 may reflect the graft’s mechanism to evade the recipient’s immune response.

Numerous questions remain unresolved, highlighting the need for further research on the mechanisms, risk factors, and biomarkers associated with ICPI-mediated rejection. This research is crucial for the development of safe protocols for the pre-and post-transplant administration of ICPIs. In addition, the importance of a washout period for pre-transplant ICPI use raises another argument for LDLT. Unlike DDLT, LDLT is a scheduled operation that allows for optimal timing and coordination of the neoadjuvant treatment protocols alongside the transplant procedure.

Munker and DeToni examined 14 verified cases of LT recipients who underwent sequential treatment with ICPIs. They found that organ vulnerability to rejection was primarily influenced by three factors: the type of immunosuppressive medication used, the PDL-1 status in liver graft biopsies, and the timing of treatment initiation. Notably, only 4 out of the 14 cases (28%) experienced liver graft rejection, with the median onset occurring within 3 weeks of starting immune treatment. Survival outcomes were available for 12 cases, with a median duration of 1.2 months.

Rammohan et al. reported a case of HCC that developed in the lung 3 years after an initial LDLT. Initially unresponsive to sorafenib, the patient showed a remarkable response to pembrolizumab, an ICPI administered at 200 mg for 21 days alongside sorafenib. After 10 months on this combined regimen, the patient exhibited stability with no detectable or radiological signs of tumor or graft rejection. A different case series by De Bruyn et al. documented a cohort of 19 LT patients treated with ICPIs for advanced malignancies. Following this treatment, 21% of the patients exhibited stable disease, while less than 38% experienced graft rejection. In a separate retrospective analysis, Abdel-Wahab et al. assessed 39 patients who had undergone allograft transplantation and found that 11 out of the 39 (28%) progressed to LT. The median time from the initiation of ICPI treatment, which included both anti-PD-1 and anti-CTLA-4 therapies, was 9 years following LT. Among these patients, only 4 out of 11 (41%) reported allograft rejection.

While data from individual reports are insufficient to conclude the superiority of one ICPI or immunosuppressant over another, several protocols have been proposed for further investigation. These include routine biopsies of liver allograft tissue prior to the initiation of any treatment in LT recipients, trials of pretreatment with immunosuppressants when there are no contraindications, and gradual tapering of immunosuppression under careful monitoring. In addition, laboratory parameters should be evaluated, including complete blood counts, comprehensive metabolic panel, and baseline oxygen saturation, which may involve a “walking oxygen saturation” assessment to identify potential declines that could necessitate further investigations.

The use of ICPIs as a therapeutic option in the palliative context following LT is currently being studied. This can largely be attributed to the limited number of viable cases for evaluation and the lack of comprehensive research on the relationship between graft rejection and tumor response. Furthermore, there might be associated clinical variables that could increase the rejection rates similarly. Challenges remain in advancing research, mainly due to the restricted availability of prognostic biomarkers relevant to patients with HCC undergoing immunotherapy in the post-LT palliative context. The application of immunotherapy in the neoadjuvant context of transplant oncology has shown promising results and is gaining increased acceptance.

Progress in this field is hindered by limited industry investment, as the potential risks associated with ICPI-mediated rejection in transplant recipients are perceived as a threat to commercial interests. This reluctance slows down the development of comprehensive studies and the identification of viable biomarkers, impeding the establishment of standardized protocols for safe ICPI administration in this patient population.

Post-LT ICPI administration remains a high-risk strategy with rejection rates of 28%–37% and substantial potential for graft loss, even when the PD-L1 status and intensified immunosuppression are considered. Although small series suggest a possible safety signal with PD-L1 inhibitors such as atezolizumab, the overall evidence base is limited to case reports and retrospective cohorts. At present, post-transplant ICPI use should be reserved for highly select patients with urgent oncologic need after mandatory multidisciplinary discussion, pretreatment graft biopsy when feasible, and close monitoring. Prospective trials are urgently required to define safe protocols that balance tumor control with preservation of graft function in this vulnerable population.

## 6 Biomarkers for risk stratification and patient selection

**Figure 3:**
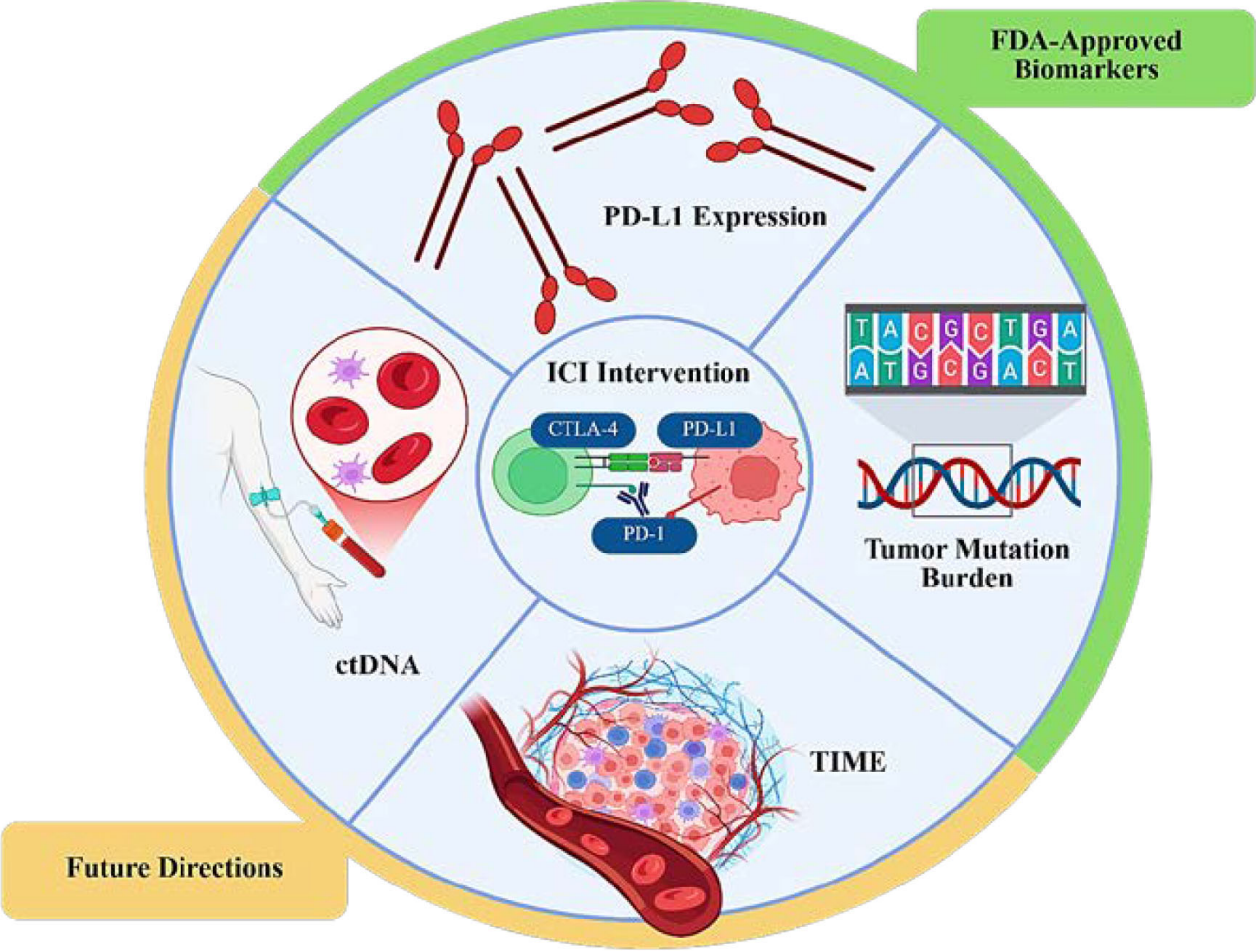
Predictive biomarkers and micro-profiling in clinical staging and treatment. FDA, Food and Drug Administration; ICI, immune checkpoint inhibitor; CTLA-4, cytotoxic T-lymphocyte associated protein 4; PD-(L)1, programmed cell death (ligand)1; ctDNA, circulating tumor DNA; TIME, tumor immune microenvironment.

Liver cancers, HCC in particular, are also highly susceptible to tumor mutation, which causes differential reactions to treatment regimens. In this context, more personalized therapeutic options are quickly becoming a priority to improve outcomes. The utilization of prognostic and predictive indications available in biomarker and mutation testing is gaining standardization to maximize clinical benefits. Among those testing for PD-L1 expression, found for optimal ICPI interventions, evaluation of the tumor immune microenvironment (TIME), ctDNA, and tumor mutation burden (TMB) analysis are predominant (**Figure 3**).

Multiple studies have documented the efficacy of ctDNA monitoring for prognostic assessment following resection and for surveillance after therapy in solid tumors, such as breast and colorectal malignancies (80, 81). Increasing data support the prognostic significance of ctDNA monitoring in patients with HCC undergoing curative liver resection (82, 83). Nevertheless, there is scant data on patients with primary liver malignancies undergoing curative LT. Two presentations from China at the 2022 American Society for Clinical Oncology (ASCO) Annual Meeting retrospectively examined the efficacy of ctDNA testing peri-LT for patients with HCC (84–86). The initial study conducted by Huang et al. assessed ctDNA fingerprinting for minimal residual disease (MRD) identification in 74 patients with HCC who had LT (84). Both pre- and post-transplantation ctDNA positivity correlated with an increased recurrence rate and diminished RFS (pre-transplantation: HR = 3.25, *p* = 0.019; post-transplantation: HR = 4.26, *p* = 0.010) (84). Furthermore, a reduction in ctDNA from pre- to post-transplantation correlated with positive clinical results. In the second study conducted by Jiang et al. (*N* = 45) (86), pre-transplantation ctDNA positivity was significantly correlated with post-transplantation recurrence (48.6% compared with 0% for ctDNA-negative patients) and a reduced disease-free survival (DFS) (390 days for the ctDNA-positive cohort *versus* not reached for the ctDNA-negative cohort, *p* < 0.05) (85, 86). These investigations indicate a possible clinical application for ctDNA testing before LT, as the ctDNA status may assist in identifying individuals who are most likely to benefit from the procedure. Notably, these investigations included ctDNA testing in the monitoring context, where a positive ctDNA result could suggest potential micrometastasis and influence treatment management decisions. Nonetheless, numerous investigations have indicated that these biomarkers exhibit inadequate sensitivity and specificity (87). Increased AFP levels may be detected in various non-HCC conditions, including pregnancy, chronic hepatitis, cirrhosis, and other cancers (intrahepatic cholangiocarcinoma, metastatic colon cancer, and germ cell tumors). Similarly, CA19–9 is frequently elevated in instances of cholangitis and common bile duct stones (88). The deficiencies of the presently utilized biomarkers underscore the need for improved prognostic biomarkers in individuals with primary liver malignancies (89).

Recently, liquid biopsy, specifically the assessment of ctDNA, has been utilized for the evaluation of TMB. The initial technology established to characterize TMB is whole-exome sequencing (WES), which analyzes non-synonymous mutations in the exomes while removing germline alterations by subtracting matched normal samples. Essential elements in this understanding include the standardization of the TMB cutoffs for categorizing patients into low, middle, and high groups, as well as methodologies for TMB calculation to guarantee consistency across various malignancies and laboratories. Various studies have suggested distinct thresholds to differentiate between high- and low-TMB groups, such as defining high TMB with a cutoff of >3 (90), >10 (91), or >20 mutations per megabase (92, 93). However, the WES approach, being technically intricate and costly, has been replaced by the emergence of next-generation sequencing (NGS) (94–96).

Numerous studies have examined the correlation between TMB and the efficacy of ICPI across various malignancies (97). The prognostic significance of TMB varies considerably among different malignancies, which is attributable to the disparate thresholds for categorizing low and high TMB values and the methodologies employed for TMB assessment in tumor tissues. HCC is ranked 12th among 30 tumors for the median number of tumor mutations (98). In the broader context of oncology utilization, a reduced TMB value has been found to correlate with an improved prognosis in renal, colorectal, and prostate cancers (99). Conversely, a lower TMB is indicative of lower survival outcomes in breast cancer, melanoma, and non-small cell lung cancer (99–101).

Collectively, while graft PD-L1 expression on pre- or post-transplant biopsy has emerged as the most clinically actionable biomarker for rejection risk stratification in the peri-LT setting, ctDNA, TIME profiling, and TMB remain largely investigational. Their current limitations, modest sensitivity/specificity, lack of HCC-specific validation, and high cost preclude their routine incorporation into transplant decision-making. In transplant oncology practice, the most practical approach combines established clinical parameters (i.e., AFP dynamics, radiological response, and explant pathology) with selective use of graft PD-L1 immunohistochemistry when biopsy is feasible. Prospective studies integrating multi-omic biomarkers with standardized washout and immunosuppression protocols are urgently needed to move beyond descriptive associations toward truly personalized risk stratification and patient selection for ICPI-based strategies.

## 7 Discussion and future directions

The conceptual framework presented above, which balances antitumor immunity against allograft tolerance through a temporally regulated process, serves as the central organizing principle of this review. By structuring the evidence around the three phases (pre-LT activation → washout → post-LT immunosuppression intensification), we highlight how timing, biomarkers, and immunosuppression intensity interact to determine both oncologic benefit and graft safety.

LT offers a potential cure for select patients with HCC and certain cholangiocarcinoma (CCA), offering the dual benefit of removing the malignancy and treating the underlying liver disease. In recent years, ICPIs—particularly PD-1/PD-L1 and CTLA-4 blocking antibodies—have revolutionized the systemic therapy for advanced liver tumors, yielding significant tumor responses and survival benefits in non-transplant settings. This has raised interest in the use of ICPIs as a neoadjuvant (downstaging) therapy to enable LT in patients initially beyond the transplant criteria. However, the deployment of ICPIs in the transplant context is controversial due to safety concerns: immune activation might precipitate acute graft rejection, given that the PD-1/PD-L1 pathways normally promote allograft tolerance.

The safety outcomes following ICPI therapy in the pre-transplant setting have varied significantly. Early reports of pre-transplant ICPI use noted serious rejection events, including fulminant graft failure, even after a long washout period. Meta-analyses of larger cohorts indicate that approximately 26% of patients who received ICPIs before LT developed acute rejection, with a 10% associated mortality. Rejection tended to occur early and ranged from mild to severe, suggesting that, while rejection is common, the outcomes may still be salvageable with prompt immunosuppressive management. Post-transplant ICPI use appears to carry an even greater risk, with graft rejection occurring in nearly 30% of cases and fatal rejection in 13%–14%. Notably, patients maintained on robust immunosuppressive regimens appear to experience fewer AEs. While a number of recipients have demonstrated positive tumor responses, these benefits must be carefully weighed against the potential for graft loss.

The timing of ICPI administration relative to transplantation significantly influences safety. ICPIs have long half-lives and lingering immune effects, raising concern about the optimal washout period. While early reports recommended a maximum 3-month interval to minimize rejection, emerging data suggest that washouts as short as 6 weeks may be feasible in select patients. The risk appears to decrease with each additional week of washout, although rare cases of late rejection have occurred. LDLT offers a unique advantage in this context by allowing precise scheduling of transplantation after an adequate washout period.

In the context of axillary liver cancers, CCA and its variants take secondary incidence and therefore, behind HCC, represent a significant corner of research studies. Current research on Cholangiocarcinoma (CCA) patients in the transplant has shown mixed results depending on the staging of the cohort evaluated. More specifically, early results for LT in locally advanced perihilar cholangiocarcinoma (pCCA) were unsatisfactory, with retrospective studies indicating elevated recurrence rates and limited survival duration (102). The incorporation of RT-based neoadjuvant therapy significantly enhanced outcomes, with studies indicating approximate 3-year recurrence rates of 25% and survival rates of 65% (103). Subsequent randomized controlled trials (RCTs) have examined the efficacy of combined stereotactic therapy in unresectable CCA, yielding good outcomes. The Advanced Biliary Tract Cancer (ABC)-01, ABC-02, and ABC-03 investigations encompassed 72 patients with pCCA, demonstrating a median PFS of 8.4 months, a median OS of 12.2 months, and an objective response rate (ORR) of 18.4% (104). The TOPAZ-1 trial randomized 685 patients with biliary tract cancer to receive gemcitabine/cisplatin with or without durvalumab, resulting in median OS of 12.9 and 11.3 months, respectively (105). The KEYNOTE-966 trial randomized 1,069 patients with biliary tract cancer to receive gemcitabine/cisplatin with or without pembrolizumab, resulting in median OS of 12.7 and 10.9 months, respectively (106). These results indicate that the implementation of multimodal systemic therapies in the pre-LT context may yield favorable outcomes for patients with pCCA who do not receive RT, as recently evidenced in other malignancies. Considering the progress in neoadjuvant therapy for many malignancies, it is appropriate to evaluate the function of radiation in the neoadjuvant context for pCCA and to anticipate that certain individuals may obtain comparable results without its inclusion.

Biomarker development is a critical area of research aimed at improving patient selection for peri-LT ICPI therapy. Graft PD-L1 expression has emerged as a potential predictor of rejection risk, with high PD-L1 expression associated with higher rates of rejection following ICPI therapy. In contrast, patients with PD-L1-negative grafts appear to tolerate ICPIs better. However, the PD-L1 status is not universally predictive, and late induction of PD-L1 or variations in expression across tumor and graft tissues complicate interpretation. Further investigation into donor-specific antibodies, T-cell profiles, and immune gene expression may yield more reliable biomarkers for predicting tolerance or rejection in the ICPI–transplant context.

Liver staging plays a fundamental role in determining transplant eligibility and guiding treatment strategies in patients with liver malignancies. Traditional staging systems such as the BCLC classification and the MC have been widely used to assess tumor burden and liver function. The MC, which limit transplantation to patients with a single tumor ≤5 cm or up to three tumors each ≤3 cm without MVI or extrahepatic spread, have demonstrated excellent post-transplant outcomes and remain the gold standard in many regions. However, newer models such as the UCSF criteria, Up-to-7, and Metroticket 2.0 expand these boundaries by incorporating additional factors such as the AFP levels and the tumor response to therapy. Dynamic assessment tools including the New York/California (NYCA) score further refine candidate selection by evaluating temporal changes in the tumor biology and the response to neoadjuvant treatment. The Risk Estimation of Tumor Recurrence after Transplant (RETREAT) score, while applied post-transplant, aids in prognostication and surveillance by incorporating the histological tumor burden, MVI, and AFP at the time of transplantation. These evolving staging systems are essential for the identification of patients most likely to benefit from LT while minimizing recurrence risk, particularly in the context of incorporating immunotherapy into transplant protocols.

Combination therapies are increasingly employed to enhance tumor control before LT or to manage recurrence afterward. Neoadjuvant use of atezolizumab plus bevacizumab has shown remarkable success in downstaging HCC, with a recent study reporting an 82% downstaging rate and no severe rejection events post-transplant when a washout period was observed (60). Similarly, the combination of ICPIs with TACE or radioembolization has yielded encouraging tumor responses. In select cases, dual checkpoint blockade or ICPIs plus tyrosine kinase inhibitors have been used safely before LT. Post-transplant, these combinations must be approached with caution due to compounded risks of rejection.

Despite progress, significant gaps in knowledge remain. There is a lack of prospective, randomized trials specifically evaluating ICPI use in transplant candidates and recipients. Mechanistic insights into ICPI-induced rejection are limited, and further biomarker development is needed to guide therapy. In addition, while the majority of data pertain to HCC, little is known about the safety and efficacy of ICPIs in transplant settings for CCA. Long-term outcomes following ICPI exposure and transplantation are also unclear, including potential impacts on chronic rejection or late allograft dysfunction.

This review, framed through the unifying conceptual model of balancing antitumor immunity against allograft tolerance across three temporally regulated phases, synthesizes the most recent multicenter cohorts and meta-analyses while highlighting the unique transplant oncology perspective. Although substantial progress has been made in understanding the washout timing, rejection mechanisms, and biomarker utility, critical gaps persist: the absence of prospective randomized trials, inconsistent immunosuppression regimens, and limited data on long-term graft function. LDLT offers the practical advantage of precise scheduling; nevertheless, even in this setting, individualized multidisciplinary decision-making remains paramount. Future efforts must prioritize well-designed clinical trials that integrate modern systemic therapies, refined biomarkers, and standardized protocols to safely expand the role of ICPIs in LT. Only through such rigorous investigation can we transform the current cautious approach into evidence-based, personalized transplant oncology care.

## 8 Conclusion

Immunotherapy has rapidly moved from the treatment of advanced HCC to earlier, potentially curative settings. In the context of LT, ICPIs offer a promising tool for downstaging and bridging patients who would otherwise remain beyond the conventional criteria. When integrated with LRTs, these can enhance tumor control, reduce waitlist dropout, and improve the pathologic response rates. However, their peri-LT application requires meticulous risk–benefit assessment, an adequate washout period, individualized immunosuppression, and close multidisciplinary monitoring.

Viewed through the unifying conceptual framework of balancing antitumor immunity against allograft tolerance across three temporally regulated phases, the available evidence supports cautious optimism. While recent multicenter cohorts suggest that neoadjuvant ICPI use is feasible in carefully selected patients, the rejection rates remain clinically an issue. LDLT provides a clear logistical advantage by enabling precise timing; however, even in this setting, long-term graft and oncologic outcomes further investigations.

Biomarker-driven selection, particularly graft PD-L1 expression, ctDNA monitoring, and dynamic AFP response, will be essential to refine candidacy and personalize therapy. Prospective randomized trials are now urgently needed to define optimal washout intervals, immunosuppression protocols, and the role of ICPIs in HCC and CCA. Only through such rigorous investigation can we safely expand the use of ICPIs in transplant oncology and realize their full potential to convert previously incurable diseases into curable ones.

This review, framed by the transplant oncology perspective, synthesizes current evidence and highlights the critical path forward. With continued advances in biomarkers, donor strategies, and well-designed clinical trials, the field stands on the threshold of meaningfully expanding curative options for patients with advanced liver cancer.
